# Mitral autograft for partial tricuspid valve replacement: a case report

**DOI:** 10.1093/jscr/rjaf057

**Published:** 2025-02-16

**Authors:** Yassin El Mourabit, Mohammed Tribak, Abderahmane El Bakkali, Lahcen Mermade, Said Moughil

**Affiliations:** Cardiovascular Surgery Department B, Hospital Ibn Sina, University Med V, Rabat 1005, Morocco; Cardiovascular Surgery Department B, Hospital Ibn Sina, University Med V, Rabat 1005, Morocco; Cardiovascular Surgery Department B, Hospital Ibn Sina, University Med V, Rabat 1005, Morocco; Cardiovascular Surgery Department B, Hospital Ibn Sina, University Med V, Rabat 1005, Morocco; Cardiovascular Surgery Department B, Hospital Ibn Sina, University Med V, Rabat 1005, Morocco

**Keywords:** mitral autograft, tricuspid valve repair, conservative surgery for infective endocarditis, infective endocarditis

## Abstract

Surgical management of tricuspid valve infective endocarditis is controversial. We report a case of infective endocarditis of the mitral and tricuspid valves which was treated by partial replacement of the tricuspid valve with a mitral autograft and replacement of the mitral valve with a mechanical prosthesis. Indication for surgical treatment was persistent tricuspid valve vegetations >20 mm after recurrent pulmonary emboli. After an 18-month postoperative follow-up, the patient was in good clinical condition with no residual tricuspid regurgitation. Mitral autograft replacement of the tricuspid valve expands the possibilities for tricuspid valve repair in the context of infective endocarditis.

## Introduction

Surgical strategies for tricuspid valve infective endocarditis include valve repair and valve replacement [1]. Tricuspid valve replacement is indicated when repair is not feasible or has failed. Currently, tricuspid valve replacements are primarily performed with bioprostheses; conversely, mechanical prostheses, while durable, pose an increased risk of thrombosis due to lower pressures and slower flows in the right heart chambers [2].

In the light of these concerns, we decided to partially replace the tricuspid valve with a mitral autograft.

## Case report

A 60-year-old man was presented with dyspnea, fever, and general deterioration. Physical examination revealed signs of heart failure and multiple rashes.

The electrocardiogram showed sinus tachycardia at 105 beats per minute.

Biological tests indicated leukocytosis with a white blood cell count of 21.250/mm^3^, elevated C-reactive protein (CRP) levels at 233 mg/L, and a markedly high procalcitonin value of 23 ng/ml. Blood cultures were negative.

**Figure 1:**
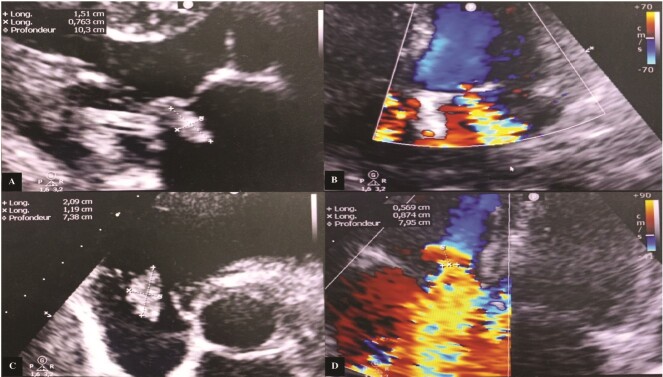
Transthoracic echocardiography images. (A) Vegetation attached to the atrial side of the anterior mitral leaflet measuring 15 × 7.6 mm. (B) Significant mitral regurgitation. (C) Vegetation attached to the free edge of the anterior tricuspid leaflet measuring 21 × 12 mm. (D) Massive tricuspid regurgitation.

Transthoracic echocardiography (TTE) revealed a mitral valve with moderate, eccentric, double-jet regurgitation, perforation of the anterior mitral leaflet, and the presence of vegetation on the atrial side of the anterior mitral leaflet, which was pedicled, mobile, and measuring 15 × 7.6 mm. The tricuspid valve had vegetation interfering with valve closure, measuring 21 × 12 mm, causing significant central tricuspid regurgitation, with a systolic pulmonary artery pressure (SPAP) at 64 mmHg (Fig. 1).

**Figure 2:**
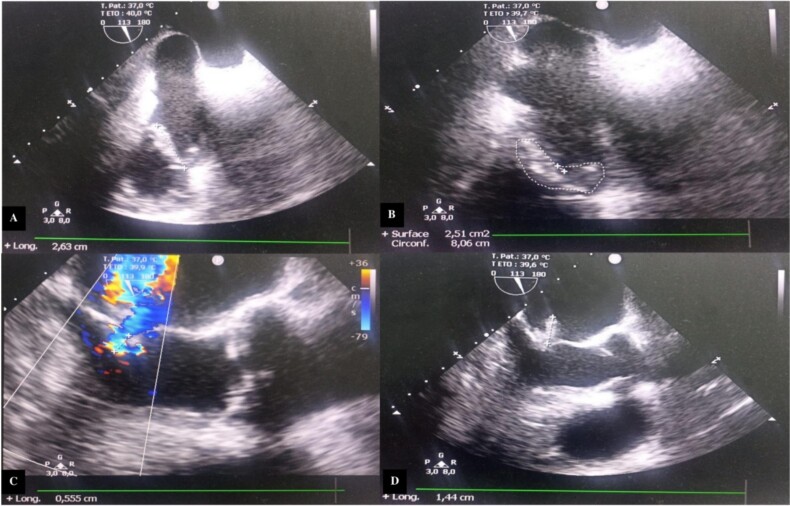
Transesophageal echocardiography images. (A) Incidence 113° showing vegetation attached to the free edge of the anterior tricuspid leaflet measuring 26.3 mm in long axis. (B) Surface area of the vegetation attached to the anterior tricuspid leaflet estimated at 2.51 cm^2^ and circumference of 8.06 cm. (C) Color Doppler showing significant mitral regurgitation. (D) Incidence 113° showing vegetation attached to the large mitral leaflet measuring 15 mm in long axis.

Transesophageal echocardiography (TEE) showed a mitral valve with vegetation prolapsing into the left atrium and a tricuspid valve with vegetation measuring 26 mm in its largest dimension, prolapsing into the right atrium (Fig. 2).

A thoraco-abdomino-pelvic computed tomography (CT) scan revealed bilateral pulmonary embolism and spondylodiscitis.

Intravenous antibiotic therapy was initiated, including vancomycin, amikacin, and targocid.

Follow-up echocardiography showed persistent vegetations and progressive worsening of valvular lesions. After 2 weeks of medical treatment, the patient was referred for surgery due to ongoing vegetations and recurrent emboli.

The procedure utilized cardiopulmonary bypass and aortic clamping. Surgical exploration of the mitral valve revealed large vegetation (20 mm in diameter) on the A3 segment of the anterior leaflet with ruptured chordae. We decided to perform a vegetectomy and resect the anterior mitral leaflet along with its subvalvular apparatus, replacing it with a mechanical mitral prosthesis.

At the tricuspid valve, there was annular dilatation, and the anterior leaflet was mutilated with large vegetation on its free edge. After removal of the infected tissue, we decided to replace the anterior leaflet with a mitral autograft that appeared non-infected.

**Figure 3:**
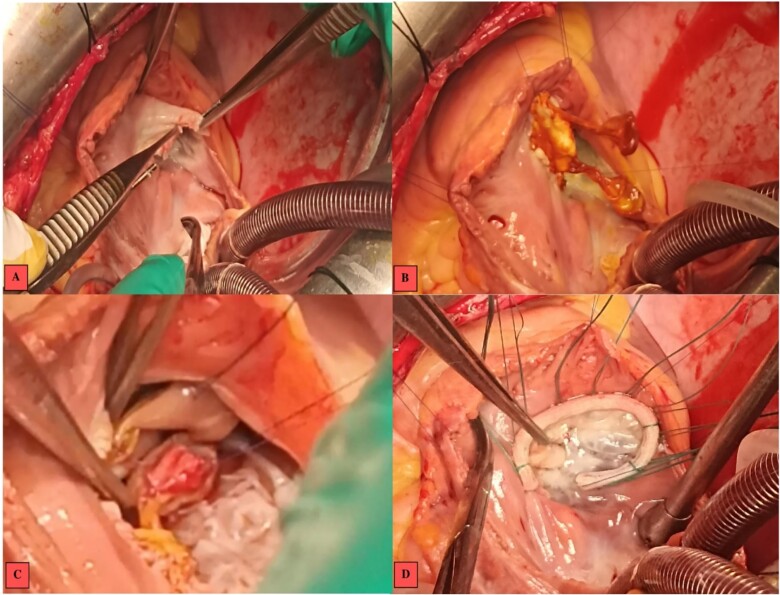
Intraoperative images. (A) Vegetations on the anterior tricuspid leaflet. (B) Valvular suture of the mitral autograft to the native tricuspid annulus. (C) Suture of the anterior papillary muscle of the mitral autograft to the interventricular septum. (D) Tricuspid annuloplasty ring with no residual regurgitation at serum testing.

**Figure 4:**
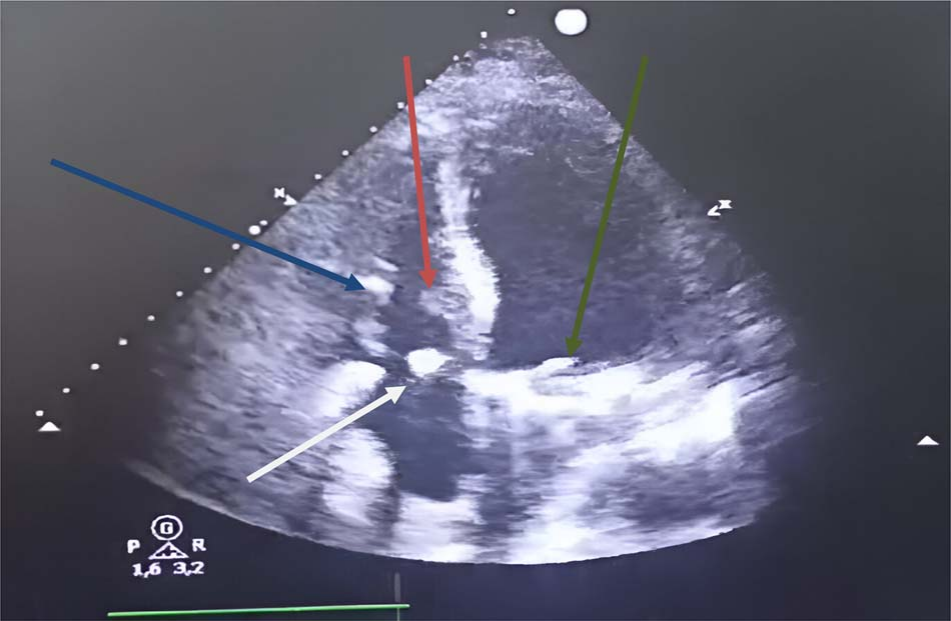
Postoperative transthoracic echocardiography, four chambers showed at 1 month. Green arrow: mechanical mitral prosthesis in place without stenosis and without detectable leakage. White arrow: tricuspid coaptation without detectable leakage, with slight excess fabric at the suture line on the anterior tricuspid leaflet. Blue arrow: inferior tricuspid papillary muscle. Red arrow: anterior tricuspid papillary muscle.

We performed an annular suture, followed by suturing the papillary muscles into the right ventricle: the anterior papillary muscle to the interventricular septum and the posterior papillary muscle to the posterior tricuspid papillary muscle. The repair was consolidated with a tricuspid annuloplasty ring (size 32; Carpentier-Edwards) (Fig. 3). Cultures of the resected segments were negative**.** Post-operative follow-up was uneventful, and echocardiographic follow-up at discharge showed no residual tricuspid regurgitation or stenosis (Fig. 4).

At the 18-month follow-up, the patient was in NYHA class I, and TTE showed mild tricuspid regurgitation. The mean pulmonary artery pressure had decreased to 18 mmHg.

## Discussion

Tricuspid endocarditis accounts for 5%–10% of infective endocarditis. It occurs in a favorable context of malnutrition, cardiac implantable electronic device and polyvalvular infectious disease [3, 4].

The treatment of tricuspid infective endocarditis is primarily medical ∼90% [5]. According to the ESC 2023 guidelines, surgical indications include persistent bacteremia for more than 7 days despite appropriate antibiotic therapy, tricuspid vegetation >20 mm that persists after recurrent pulmonary emboli, involvement of left-sided structures, or respiratory insufficiency requiring ventilatory support after recurrent pulmonary emboli [6].

Regarding the timing of surgery, we recommend the strategy of operating at an early stage in the evolution of the infectious process [7]. This approach aims for successful repair before advanced destruction of the valve occurs [8].

The feasibility of repair depends on infection extent and remaining healthy tissue [9]. When the quality and integrity of the tissue allow it, repair is preferable to replacement [10]. However, the repair rate remains lower than the replacement rate, due to advanced valvular lesions making repair uncertain after excision of infected tissues, and the surgeon’s experience.

Mortality varies little by technique, but repair has lower perioperative complications. In contrast, valve replacement results in longer stays in intensive care and the hospital, as well as higher hospital costs [11]. Additionally, repair is linked to lower rates of endocarditis recurrence and reintervention, with no significant difference in long-term all-cause mortality [12].

Using a mitral autograft rather than a tricuspid homograft is justified by the better quality of mitral tissue, which is easier to manipulate [13]. Some authors base their approach on inserting the autograft with the anterior leaflet oriented toward the septum and the papillary muscles externalized and sutured to the wall of the right ventricle [14]. Insertion of the mitral autograft in the tricuspid position can be achieved using a “buttonhole” technique for the two papillary muscles of the autograft [15].

In our patient, we proceeded by suturing the mitral autograft leaflet tissue to the residual native leaflet tricuspid, followed by suturing the papillary muscles into the right ventricle: the anterior papillary muscle to the interventricular septum and the posterior papillary muscle to the posterior tricuspid papillary muscle.

Once the mitral leaflet was in place, we pulled its edge against the native septal leaflet in the systolic position to determine the implantation site of the papillary muscle on the free wall of the right ventricle, which did not correspond to the native tricuspid papillary muscle sites.

This technique allows more flexibility in positioning the mitral autograft in the right ventricle, taking into account the anatomical differences between the subvalvular apparatus of the mitral and tricuspid valves.

Finally, we considered it necessary to insert a prosthetic tricuspid remodeling ring because of tricuspid annular dilatation.

## Conclusion

Tricuspide valve repair using mitral autograft should be considered in cases of infectious tricuspid insufficiency due to the expected benefits in terms of morbidity and mortality.

Long-term follow-up is required to evaluate clinical results and compare hemodynamic performance with that of bioprostheses.
